# 3D‐Printed Structures Versus Drilled Cavities: A Comparison of Microconfinement Methods for Rheological Characterisation of Multicellular Aggregates

**DOI:** 10.1002/jmr.70040

**Published:** 2026-07-02

**Authors:** Isis V. M. Lima, Chukwuma Chris Muoghalu, Shruti G. Kulkarni, Mènie Wiemer, Jonas Michalewski, Sander van den Driesche, Wiebke Gehlken, Michael J. Vellekoop, Manfred Radmacher

**Affiliations:** ^1^ Institute for Biophysics, University of Bremen Bremen Germany; ^2^ Institute for Microsensors, ‐Actuators and ‐Systems (IMSAS), University of Bremen Bremen Germany; ^3^ MAPEX Center for Materials and Processes University of Bremen Bremen Germany; ^4^ Microsystems Center Bremen (MCB) University of Bremen Bremen Germany

**Keywords:** 3D‐printed truncated conical microstructures, atomic force microscopy, cylindrical cavities, human pancreatic cancer cells, microconfinement, multicellular aggregates, PolyHEMA, viscoelasticity

## Abstract

Three‐dimensional (3D) cell culture systems are increasingly recognised as more physiologically relevant models than traditional two‐dimensional cultures, as they better mimic the native tumour microenvironment and enable the study of complex cellular behaviour. However, applying atomic force microscopy (AFM) to these models is challenging due to the inherent instability of multicellular aggregates, which complicates reproducible mechanical property measurements. To address this, we developed and evaluated two distinct strategies for confining multicellular aggregates for AFM analysis. In the first approach, aggregates were encapsulated in porous 3D‐printed truncated conical microstructures fabricated by two‐photon polymerisation. These structures were designed to allow medium perfusion, which is hypothesised to improve nutrient and metabolite exchange by enhancing fluid accessibility within the confined environment. In the second, cylindrical cavities were microfabricated into the base of PolyHEMA‐coated Petri dishes to provide a simple yet robust platform for aggregate retention. Both methods successfully confined aggregates without compromising cellular integrity, enabling reproducible and reliable measurements of stiffness and viscoelasticity. Human pancreatic cancer cells (PANC‐1) were used both as single cells and as monotypic aggregates. Aggregates confined in either system were consistently softer than substrate‐attached single cells, indicating reduced cytoskeletal tension and altered remodelling in 3D environments. Among the two approaches, cylindrical cavities provided more reliable aggregate retention, whereas the 3D‐printed truncated conical structures may possibly facilitate improved medium access through their porous architecture. Together, these microconfinement strategies enable reproducible mechanical characterisation of multicellular aggregates and extend the applicability of AFM to tumour mechanobiology and the assessment of anticancer therapies.

## 1 Introduction

Cancer cells do not exist in isolation. Within the organism, they are embedded in complex tissues and continuously interact with their surrounding microenvironment [1]. These interactions are fundamental to tumour growth, progression, and therapeutic response. As demonstrated by Kapałczyńska et al. [2], conventional two‐dimensional (2D) cultures, although widely used, fail to reproduce the structural and physical cues experienced in vivo. Ravi et al. [3] emphasised that three‐dimensional (3D) culture systems overcome this limitation by performing more accurately the cell–cell and cell–matrix interactions [4]. The concept of modelling tumour growth in three dimensions was pioneered by Sutherland et al. [5], who developed the multicellular tumour spheroid as a scaffold‐free in vitro model of nodular carcinomas, thereby establishing the foundation for modern 3D cancer research. The importance of the microenvironment's biophysical properties was highlighted by Discher et al. [6], who demonstrated that cells actively sense and respond to matrix stiffness, triggering changes in their cytoskeletal organisation and altering their behaviour. Subsequent work by Mih and Horkay [7] and Levental et al. [8] further showed that tissue stiffness and elasticity directly regulate cancer cell proliferation, migration, and invasion. To capture these effects quantitatively, rheological approaches are increasingly employed to assess not only stiffness but also the viscoelastic properties of cells and tissues, distinguishing their elastic (solid‐like) from their viscous (fluid‐like) responses [9]. Both passive methods, such as particle‐tracking microrheology, and active methods, such as micropipette aspiration or magnetic tweezers, have been used. In contrast, atomic force microscopy (AFM) applies well‐defined forces with nanometre precision, enabling quantitative mapping of mechanical properties in both individual cells [10] and multicellular structures. Even though in most AFM studies only the elastic properties are determined, new modes have been developed to characterise the viscoelastic properties of soft samples by AFM [9, 11, 12].

Early AFM studies showed that cancer cells are softer than non‐cancerous cells [13], a feature that may facilitate metastatic potential [14, 15]. However, it has been shown that cell–matrix adhesions differ significantly between two‐dimensional and three‐dimensional environments, influencing cell mechanics and behaviour [16]. More recently, AFM has been applied to living multicellular aggregates and spheroids, enabling the investigation of tumour models in physiologically relevant conditions. In these models, spheroids are typically generated in agarose‐coated wells, usually supplemented with extracellular matrix (ECM) components such as collagen, and then transferred to Petri dishes. While this approach preserves spheroid viability, it does not fully prevent mechanical instability, as spheroids remain susceptible to partial dissociation during handling, with individual cells reattaching to the substrate and disrupting the aggregate. Several strategies have been explored to address this issue, each presenting specific limitations. For instance, encapsulation within hydrogels prevents substrate adhesion but may restrict nutrient diffusion and alter cell behaviour [17]. Microwell arrays and engineered surfaces improve containment but often limit accessibility or require complex fabrication processes [18]. Even the common practice of culturing spheroids in multi‐well plates and subsequently transferring them to Petri dishes for AFM introduces variability and increases the risk of mechanical damage. These challenges are particularly relevant when studying viscoelastic properties at a collective level. Abidine et al. [19] examined bladder cancer spheroids and found that their mechanical response differed from that of isolated cells, with behaviour strongly influenced by the surrounding collagen environment. These findings highlight how multicellular organisation and the extracellular matrix can give rise to emergent mechanical features not apparent in single‐cell measurements. Similarly, Taubenberger et al. [20] showed that spheroid stiffness is strongly modulated by the rigidity of the surrounding hydrogel microenvironment, while Anselmetti et al. [21] reported marked spatial heterogeneity within spheroids, with stiffer outer regions and softer inner cores. Efremov et al. [22] further demonstrated that the overall stiffness of a multicellular aggregate cannot be predicted from individual cell properties alone but instead arises from complex interactions between cell junctions [23]. Moreover, as Langhans [24] emphasises, three‐dimensional in vitro models, such as spheroids and extracellular matrix‐embedded aggregates, capture mechanical and microenvironmental cues absent in conventional two‐dimensional systems, improving the physiological relevance of drug discovery and mechanical characterisation.

**FIGURE 1:**
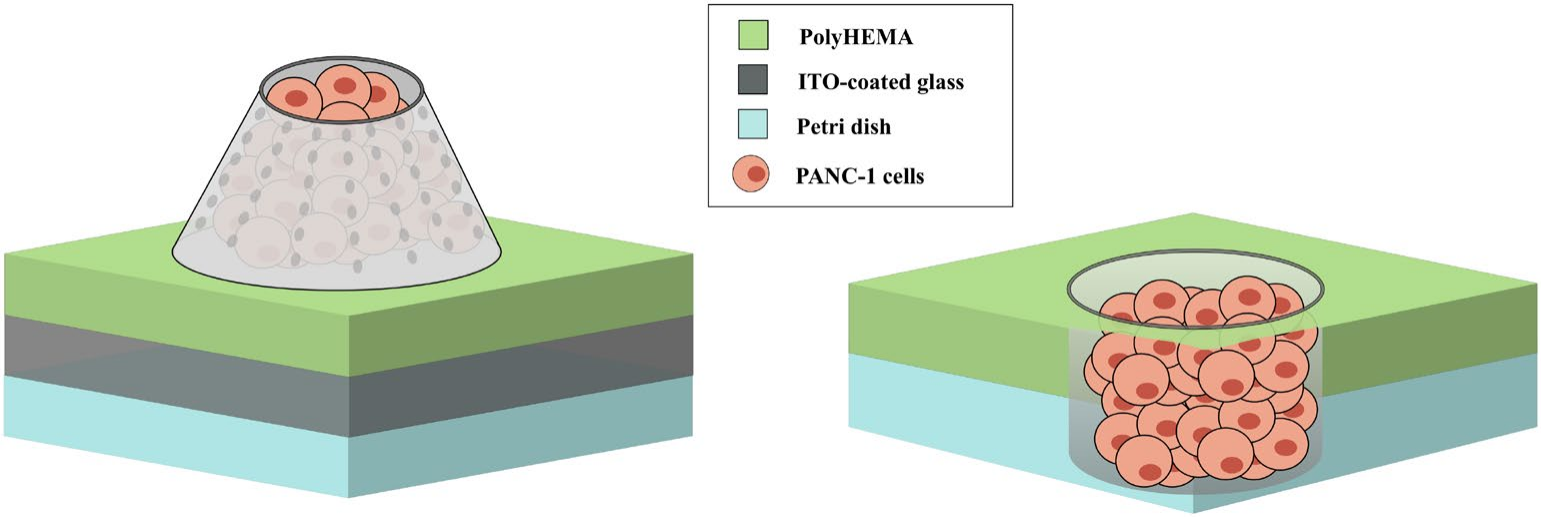
Illustration of the two confinement strategies used for probing PANC‐1 multicellular aggregates with AFM. Representative schematics of the two confinement approaches used for PANC‐1 multicellular aggregates. On the left, a schematic design of the truncated cone structure employed in this work is shown. The structure was first 3D‐printed on an ITO‐coated glass substrate (grey). The ITO support, containing the printed truncated cones, was then glued to the Petri dish (blue) and subsequently coated with a PolyHEMA layer (green). On the right, a cylindrical cavity, described as a hole in the PolyHEMA‐coated Petri dish, represents the second confinement approach used in this work. In both configurations, PANC‐1 cell aggregates were cultured and mechanically characterised to evaluate the influence of the confinement method.

Therefore, these studies highlight the need for standardised approaches that preserve spheroid integrity [25, 26], control microenvironmental stiffness, and enable accurate mechanical measurements of multicellular aggregates. To address these limitations, we developed two complementary approaches to stabilise pancreatic cancer aggregates for AFM analysis, enabling culture and measurement within the same Petri dish. The first approach employs 3D‐printed truncated conical microstructures fabricated with two‐photon polymerisation, providing precise spatial confinement and enabling medium access within the structure. The second approach uses cylindrical cavities microfabricated into the bottom of PolyHEMA‐coated Petri dishes, a polymer that reduces cell adhesion, allowing support for cell aggregates in a three‐dimensional configuration. A comparison of these two confinement strategies is shown in Figure 1. PolyHEMA molecules polymerise when exposed to heat, forming long chains that create a stable structure under cell culture conditions for extended periods, while also being biocompatible. While PolyHEMA is commonly used to inhibit cell adhesion during spheroid formation, we adapted its use to create a specialised platform for aggregate retention. This method preserves aggregate integrity and physiological conditions by eliminating the need for transfer, a common source of mechanical stress. Hence, both methods offer distinct advantages: the 3D‐printed microstructures provide well‐defined spatial confinement and are designed to facilitate medium access within the structure, whereas the microcavities provide a straightforward, easily scalable solution for stable aggregate containment. Together, these methods address the challenges of mechanical instability and partial dissociation during handling, facilitating reproducible quantification of tissue‐level viscoelastic properties in living 3D tumour models. This study makes three main contributions. First, reliable and versatile platforms were developed for stabilising 3D aggregates during AFM measurements, addressing the limitations of conventionally used free‐floating aggregates. This improvement enhances not only reproducibility but also allows continuous or repeated measurements on the same aggregate over periods of days to weeks. Second, key insights are provided into how the design of these supporting structures influences aggregate behaviour and mechanical properties, offering practical guidance for future experimental design. Finally, the first systematic measurements of pancreatic cancer aggregate mechanics under different confinement strategies are presented. The results show that these 3D aggregates are significantly softer than adherent cells, reflecting the reduced structural tension in a three‐dimensional microenvironment. Together, these contributions offer both practical tools and critical insights for mechanobiology research, expanding the application of AFM in studying tumour mechanics under physiologically relevant conditions.

## 2 Materials and Methods

### 2.1 Preparation of the PolyHEMA‐Coated Supports

Polystyrene Petri dishes (Sarstedt, 60 × 15 mm, with ventilation cams, sterile) were coated with Poly (2‐Hydroxyethyl Methacrylate), commonly known as PolyHEMA (Sigma‐Aldrich, St. Louis, MO, USA; average molecular weight M_v_ = 300,000, crystalline). To prepare the stock solution, PolyHEMA was dissolved in 95% ethanol to obtain a concentration of 120 mg/mL [27]. The stock solution was mixed overnight at 500 rpm using a magnetic stirrer (Heidolph MR 3000 D) to ensure homogeneity. Subsequently, 2.44 mL of PolyHEMA at a working concentration of 5 mg/mL was added into a Petri dish [28]. Finally, PolyHEMA‐coated Petri dishes were obtained after the evaporation of the ethanol fraction (20 h at 37°C in a drying oven; Heraeus Instruments, Kelvitron T 6030).

### 2.2 Cell Culture

In this work, the human pancreatic carcinoma cell line, PANC‐1, was used. Cells were cultured in high‐glucose Dulbecco's Modified Eagle's Medium (DMEM; Cytion/CLS Cell Lines Service GmbH, Eppelheim, Germany) supplemented with 10% foetal bovine serum and 1% penicillin–streptomycin. They were incubated at 37°C in a humid atmosphere with 5% CO_2_. Whenever the cultures reached 80% confluency as a monolayer, cells were detached with trypsin (ROTI Cell Trypsin/EDTA, 1× solution, 0.05% in DPBS; Carl Roth GmbH & Co. KG, Germany) for 5 min in the incubator and subsequently transferred into a new flask for continued culture. Cells were used between passages 5 and 18 after being thawed and harvested.

For single‐cell AFM experiments, cells were seeded directly onto untreated polystyrene Petri dishes (Sarstedt, 60 × 15 mm, with ventilation cams, sterile) with 4 mL of supplemented DMEM to allow normal adhesion and spreading. A total of six Petri dishes were prepared as biological replicates, and AFM measurements were consistently performed 48 h after cell seeding, when cells were firmly adhered to the substrate. In total, 52 single adherent cells were measured using both conventional force‐curve measurements and sweep frequency (chirp) experiments, with approximately eight cells measured in distinct regions of each Petri dish. For aggregate formation, a Neubauer chamber was used to count PANC‐1 cells, and 300,000 cells were seeded into PolyHEMA‐coated Petri dishes containing 4 mL of complete DMEM. The non‐adhesive PolyHEMA surface prevented cell attachment to the substrate and promoted the spontaneous formation of multicellular aggregates. After four days of incubation, well‐defined aggregates were observed and subsequently transferred into the confinement structures for further culture and mechanical characterisation (see below).

### 2.3 3D‐Printed Structures: Design and Fabrication

**FIGURE 2:**
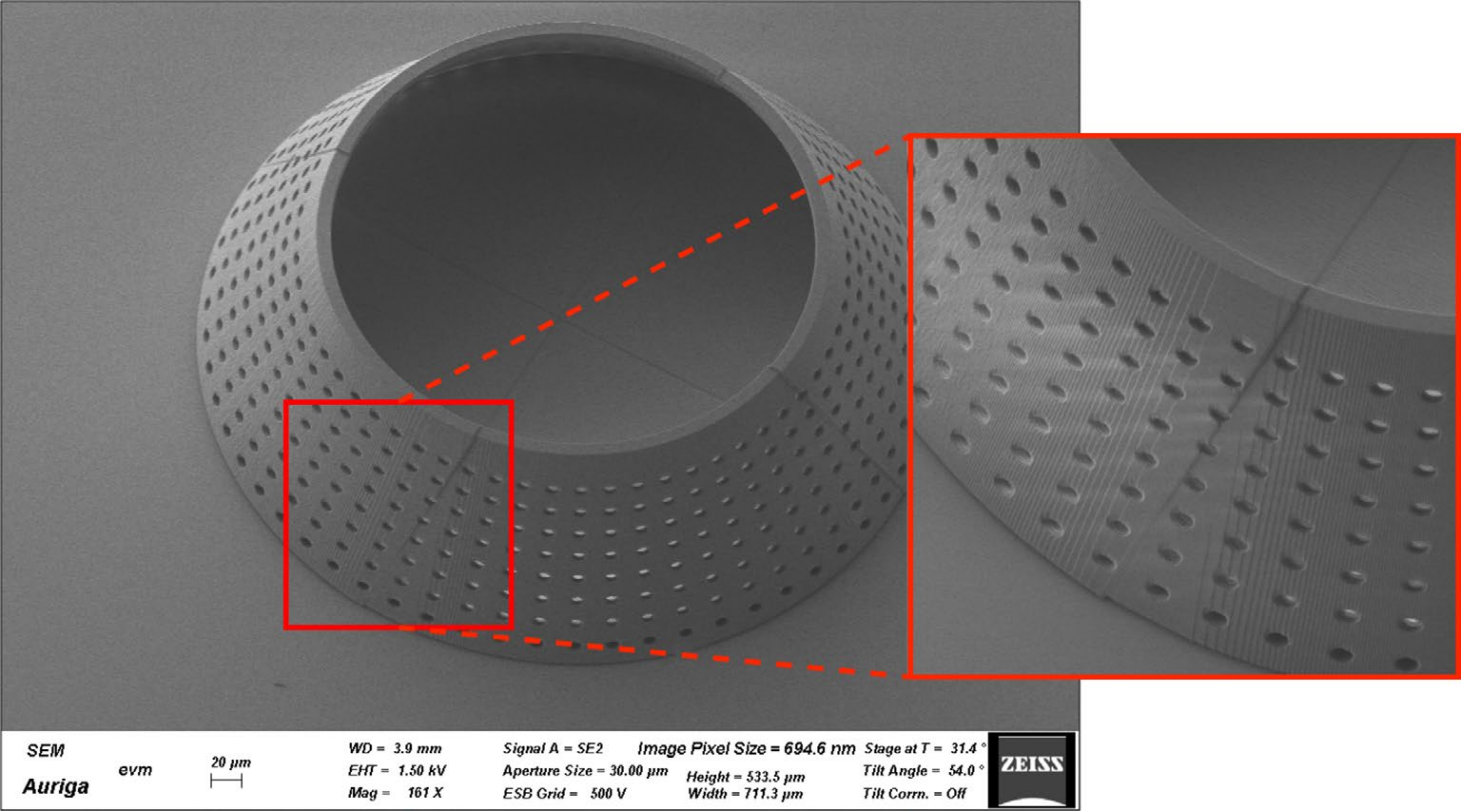
Scanning electron microscope (SEM) images of 3D‐printed truncated cone structures. Scanning electron microscope (SEM) images of 3D‐printed truncated cone, showing the overall geometry and a magnified view of its porous surface. The pore architecture is designed to allow perfusion of culture medium through the construct and is intended to support diffusion‐based transport within the structure; however, this function was not experimentally evaluated in the present study. Dimensions are as follows: Top diameter 500 μm, bottom diameter 800 μm, and height 300 μm.

3D‐printed truncated conical microstructures were designed using the software Fusion 360 (Autodesk) by employing standard geometric operations, including extrusion, revolution, lofting, and filleting tools. The designs were then exported as high‐resolution STL (Standard Tessellation Language) files for subsequent processing with DeScribe slicing software (Nanoscribe GmbH & Co. KG, Karlsruhe Germany). The corresponding design files are openly available to the community on Zenodo [29]. Printing was performed with a Nanoscribe Photonic Professional GT2 system using two‐photon polymerization (2PP), which allowed for the fabrication of features with sub‐micrometer resolution. The non‐cytotoxic photoresin IP‐Dip (ISO 10993‐5 / USP 87) was used as the printing material. The structural features of the 3D‐printed truncated cones were characterised using an Auriga 40 scanning electron microscope (SEM, Zeiss & Raith, Germany), as shown in Figure 2. High‐resolution images were acquired to observe its porous architecture, consisting of 20 μm circular channels spaced 11 μm apart, with a height of 300 μm, a top diameter of 500 μm, and a base diameter of 800 μm.

#### 2.3.1 Substrate Preparation and Mounting

The truncated cone structures were printed directly onto an ITO‐coated glass substrate and glued to a Petri dish using Hobby Fix underwater glue (Dohse Aquaristik GmbH & Co. KG, Grafschaft‐Gelsdorf, Germany), a non‐toxic adhesive providing a stable base for the experiments. After allowing the glue to dry, the surface around the printed structures was coated with PolyHEMA to prevent cell adhesion, leaving the truncated cones uncoated.

#### 2.3.2 Aggregate Transfer to 3D Microstructures

Cell aggregates were first cultivated in PolyHEMA‐coated Petri dishes. After aggregate formation, they were transferred into the 3D microstructures by gently pushing them into the top opening of the conical frustums using GELoader Tips (0.5–20 μL, Eppendorf). The pipette tips were sterilised by autoclaving at 121°C for 20 min prior to use. The inclination of the designed truncated conical geometry facilitated smooth settling of the aggregates inside the printed structures, minimising mechanical stress. The 3D‐printed microstructures are reusable and can be cleaned and sterilised using the procedure described in the Supporting Information [Methods S1], which preserves their structural integrity and reduces fabrication effort and material costs.

### 2.4 Microfabrication of Cylindrical Cavities

A milling machine was used to drill cylindrical cavities in the inner bottom of PolyHEMA‐coated Petri dishes. The dishes were coated before drilling to ensure that the cells adhered primarily to the uncoated plastic surfaces produced by the drilling process, providing an ideal environment for cell attachment and retention. The dimensions of the cavities were determined by the diameter of the drill bits used (500 and 800 μm) and by the electronic sensor integrated into the milling machine, which was set to two different depths (300 and 600 μm). Due to the absence of PolyHEMA inside the drilled cavities, the cells naturally aggregated and proliferated within the confined structures.

### 2.5 Optical Imaging of Aggregates in Truncated Cones and Cylindrical Cavities

Optical images of PANC‐1 cell aggregates on PolyHEMA‐coated Petri dishes were acquired to visualize both confinement methods. The same objective and camera were used for both images (ZEISS CP‐Achromat 5×/0.12 air objective; APTINA CMOS industrial digital camera, 8 MP) mounted on an Axiovert 25 inverted microscope (Carl Zeiss, Germany).

### 2.6 AFM Experiments

The experiments were performed using an MFP‐3D atomic force microscope (AFM) manufactured by Asylum Research (Digital Instruments, Santa Barbara, CA), integrated with an inverted optical microscope (Zeiss Axiovert 135; Zeiss, Oberkochen, Germany) with a 10× objective and 1× Bertrand lens in bright field mode. Moreover, MLCT‐BIO‐DC cantilevers (type C) with a drift‐compensated gold pad on their top side were used, resulting in a low thermal sensitivity. These probes have pyramidal tips and triangular‐shaped cantilevers. The cantilevers were made of silicon nitride (Si_3_N_4_), fabricated by Bruker Nano Surfaces and Metrology, with a nominal spring constant of *k* = 10 mN/m, an opening angle of 35°, and a resonance frequency of 7 kHz in air and 1 kHz in liquid, as determined during the calibration. Samples in Petri dishes were attached to an aluminium holder using vacuum grease and positioned on the AFM stage with two magnets.

The whole setup was enclosed in a custom‐made polymethylmethacrylate (PMMA) chamber to facilitate the injection and control of 5% CO_2_ levels, in order to maintain a stable pH in the cell culture medium. The AFM was enclosed in a wooden box lined with acoustic damping foam to minimise environmental and acoustic noise, and was lifted from the floor to reduce vibrations and other disturbances that could introduce measurement artefacts. The influence of mechanical forces and cell–matrix interactions on tumour progression has been widely recognised [30]. Guided by this concept, two complementary methods were employed to apply controlled external forces to the samples. In the first approach, conventional AFM force curves were performed to quantify the elastic properties of the samples. During each measurement, the AFM tip was brought into contact with the surface, the cantilever deflection was recorded throughout indentation, and the tip was subsequently retracted. Second, the sweep frequency method was employed to evaluate viscoelastic behaviour, whereby a sinusoidal deformation was applied to the sample through the z‐piezo actuator.

### 2.7 AFM Data Analysis

The mechanical properties of individual PANC‐1 cells and cell aggregates derived from the same cell line were characterised by analysing AFM force indentation data. IGOR Pro version 9 (WaveMetrics, Lake Oswego, OR, USA) was used for data acquisition and analysis. Force–distance curves were acquired in force mapping mode, with 10 × 10 curves per map collected from five different regions of the aggregates, including central and peripheral areas, using a scan size of 4 μm. The apparent Young's modulus was determined by fitting the approach portion of each force curve with the Hertz model Supporting Information [Figure S1]. The fit was performed within a force range of 10–500 pN, assuming a Poisson's ratio of 0.5 for biological samples, and defining the threshold contact point at 3 nm, as described by Radmacher [31]. Elasticity was represented as logarithmic histograms, and the median with interquartile ranges was considered for each force map. To assess viscoelastic properties, frequency sweep measurements were performed by applying a sinusoidal oscillation (50 nm amplitude) over a frequency range of 1–100 Hz, limited by the response of the piezoelectric ceramics. Each sweep lasted 8.7 s, with 4 × 4 curves per map collected from the same regions analysed in the elastic measurements [Figure S2a,b]. This protocol enabled consistent spatial comparison of both elastic and dynamic mechanical properties across the aggregates. These measurements were fitted using the power‐law structural damping model Supporting Information [Figure S3] [32].

This approach decomposes the complex modulus (E*) into its real (elastic or storage modulus) and imaginary (viscous or loss modulus) components. The full derivation is presented in the Supporting Information [Analysis S1]. The storage modulus reflects the energy stored and recovered during oscillatory deformation, while the loss modulus quantifies the energy dissipated per cycle of oscillation. Additionally, the loss tangent (tan δ), that is, the ratio of the loss to storage modulus, was obtained, which provides insight into whether the cell exhibits predominantly solid‐like (tan *δ* < 1) or liquid‐like (tan *δ* > 1) behaviour. In this model, the storage modulus increases with frequency according to a power‐law with exponent Supporting Information [Analysis S2], while the loss modulus comprises a fractional component of the storage modulus along with an additional Newtonian viscous term [33]. To evaluate the statistical significance of differences in Young's moduli, pairwise comparisons between groups were performed using the Wilcoxon rank‐sum test (*p* < 0.01), a non‐parametric method that does not assume normality of the data. This test provides a robust assessment of differences in median force values between single cells and cell aggregates.

### 2.8 Visualisation of Viscoelastic Properties

Several mechanical parameters were measured to quantify the properties of both single cells and cellular aggregates in different confinements. To better visualise the differences between such experimental groups of data, principal component analysis (PCA) was performed on each distinct cloud of data (storage modulus versus loss modulus). Evaluating the joint distribution via PCA provides greater clarity regarding systemic differences than comparing independent, one‐dimensional histograms.

For the PCA, the covariance matrix is computed from the logarithms of the storage and loss moduli of each force curve within a given group, that is, confined aggregates in cylindrical cavities or truncated cones. The logarithmic transformation of the moduli has been chosen, as it has previously been observed that the data exhibit a lognormal distribution. The covariance matrix is diagonalised, whereby the eigenvalues and eigenvectors of this 2 × 2 matrix are determined. The eigenvectors define an ellipse in the two‐dimensional parameter space, with the principal axes oriented along the directions of the respective eigenvectors, while the eigenvalues determine their lengths. The scaling of these lengths is chosen such that 67% of the data are enclosed within the ellipse. This is analogous to the one‐dimensional case, in which 67% of the data lie within a range of ±1*σ* around the mean, where σ represents the standard deviation.

## 3 Results

### 3.1 Mechanical Properties of Single Cells and Aggregates

The development of robust confinement strategies is crucial for the long‐term mechanical characterisation of cellular aggregates by AFM. While two‐dimensional (2D) cell cultures offer baseline measurements of cellular stiffness, cytoskeletal organisation, and substrate‐dependent adhesion, they fail to replicate the complexity of in vivo environments. On the other hand, confining aggregates within physiologically relevant three‐dimensional (3D) conditions allows researchers to investigate tissue organisation, mechanobiology, and disease progression more faithfully.

Building on this principle, recent advances in microfabrication have enabled the creation of precisely defined confinement geometries. For example, Taale et al. [34] demonstrated that two‐photon polymerisation can generate dome‐shaped confinements around multicellular spheroids, providing high spatial and temporal control over growth, migration, and actin cytoskeletal dynamics at the single‐spheroid level. In contrast, the porous truncated conical microstructures and cylindrical cavities enabled long‐term AFM measurements by stabilising multicellular aggregates under consistent in vitro culture conditions, allowing the same aggregates to be followed over extended periods while maintaining controlled confinement and medium access. Whereas the cylindrical cavities were produced by mechanical drilling, both the previously reported dome‐shaped confinements and our truncated cone structures were fabricated using the same technique with distinct designs and purposes. Importantly, our structures prioritise reproducibility and mechanobiological measurements over extended culture periods, thereby providing a practical platform for systematically probing how three‐dimensional confinement influences the mechanical and viscoelastic properties of multicellular aggregates. To investigate the influence of microenvironmental confinement on multicellular aggregate mechanics, two engineered microstructures, porous 3D‐printed truncated cones and drilled cylindrical cavities, were evaluated for their ability to retain PANC‐1 aggregates while permitting reproducible AFM measurements [35]. Long‐term characterisation was thus enabled, capturing temporal changes in aggregate mechanics associated with cellular remodelling and disease progression Supporting Information [Videos S1 and S2].

**FIGURE 3:**
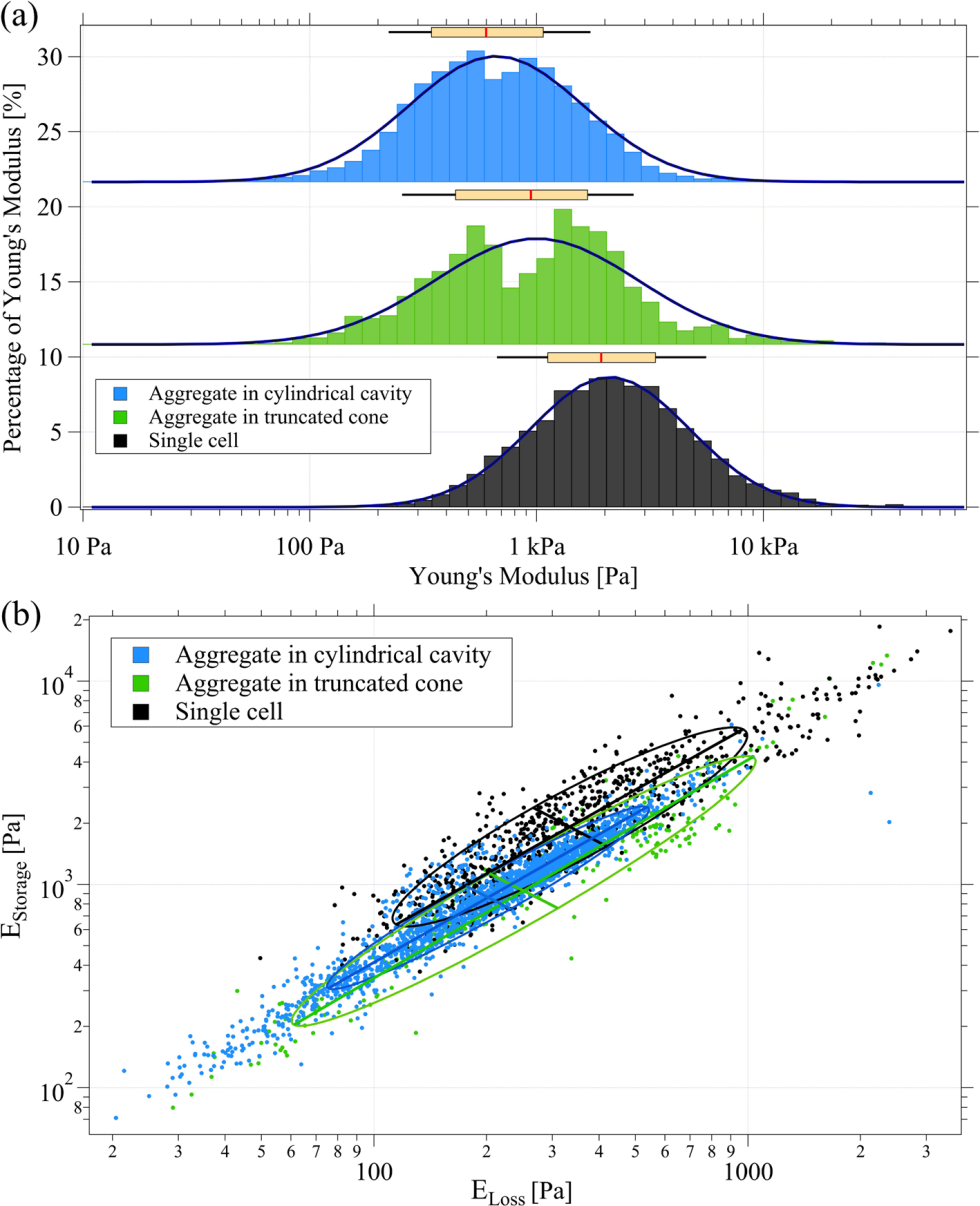
Microenvironmental confinement modulates the mechanical properties of PANC‐1 cancer aggregates. Histograms of Young's modulus obtained from AFM measurements. Single cells on uncoated Petri dishes (black), aggregates in 3D‐printed truncated cones (green), and aggregates within drilled cylindrical cavities (blue). (a) Histograms of Young's modulus (E) from AFM measurements for single adherent PANC‐1 cells on uncoated Petri dishes (black) and PANC‐1 aggregates confined in 3D‐printed truncated cones (green) or drilled cylindrical cavities (blue). (b) Principal component analysis (PCA) of the frequency‐dependent viscoelastic properties of PANC‐1 samples. The analysis was performed on the logarithm of storage and loss moduli measured from all force curves. Each point represents one independent force‐map measurement. Samples are grouped according to predefined experimental conditions (adherent single cells, aggregates formed in truncated cones, and aggregates formed in cylindrical cavities). The PCA was used to visualize the covariance structure of the dataset in a reduced two‐dimensional space (PC1 and PC2). Ellipses represent covariance‐based confidence regions of each predefined group in the PCA space, illustrating differences in mechanical variability between conditions.

Figure 3a shows histograms of Young's moduli obtained from AFM measurements of single adherent PANC‐1 cells on uncoated Petri dishes (black bars), aggregates in 3D‐printed truncated cones (green bars), and aggregates within drilled cylindrical cavities (blue bars). To minimise potential artefacts arising from direct AFM cantilever contact with loosely attached central cells, stiffness measurements were primarily performed at the aggregate periphery. Aggregates in both confinement geometries exhibited lower storage and loss moduli and slightly higher power‐law exponents compared with single adherent cells [36]. Power‐law exponents (*α*) for biological materials are typically reported in the range of approximately 0.1 to 0.5, reflecting weak power‐law viscoelasticity with intermediate elastic and viscous characteristics as follows: Supporting Information [Table S1].

#### 3.1.1 Statistical Analysis

Pairwise comparisons using the Wilcoxon rank‐sum test (see Table 1) showed that single cells generated significantly lower elastic modulus values than aggregates confined in truncated cones and cylindrical cavities (*p* < 0.01 for both comparisons). The analysis was performed using the power‐law structural damping model, which accounts for storage and loss moduli and includes hydrodynamic contributions to describe viscoelastic behaviour more accurately [37, 38]. For visualisation, Figure 3b presents scatter plots of storage modulus versus loss modulus. Black data points represent single cells adherent to the Petri dish, whereas green and blue data points correspond to aggregates formed in truncated conical and cylindrical cavities, respectively. The viscoelastic parameters (storage modulus *E'* and loss modulus *E"* across the measured frequency range) extracted from each force‐map measurement were used to construct a multivariate dataset. In the principal component analysis (PCA), each point represents an individual force‐map measurement (i.e., one analysed region of a cell or aggregate) projected into PCA space. Ellipses were fitted using principal component analysis to enclose median values of each condition, showing a clear separation between single cells and aggregates in viscoelastic space. Principal component analysis was performed to facilitate the comparison of variations in the storage and loss moduli when comparing different groups (i.e., single cells, aggregates in cylindrical cavities, and aggregates in truncated cones). From the logarithms of the moduli obtained from all force curves, the two‐dimensional covariance matrix was calculated and subsequently diagonalised by determining its eigenvalues and eigenvectors. These eigenvalues and eigenvectors were then used to define ellipses along the corresponding two major axes. The lengths of the axes are proportional to the eigenvalues and an appropriate scaling factor, resulting in approximately 67% of the data being enclosed within each ellipse.

**TABLE 1:** Wilcoxon pairwise comparisons of cells and aggregates in confined geometries.

Comparison	*p*‐value	Upper	Lower	Significance
Single cells vs. truncated cones	6.96 × 10^−8^	3.48 × 10^−8^	1	*p* < 0.01 (significant)
Single cells vs. cylindrical cavities	0	0	1	*p* < 0.01 (highly significant)
Truncated cones vs. cylindrical cavities	0.0219	0.011	0.989	*p* > 0.01 (not significant)

### 3.2 Long‐Term Stability and Retention

**FIGURE 4:**
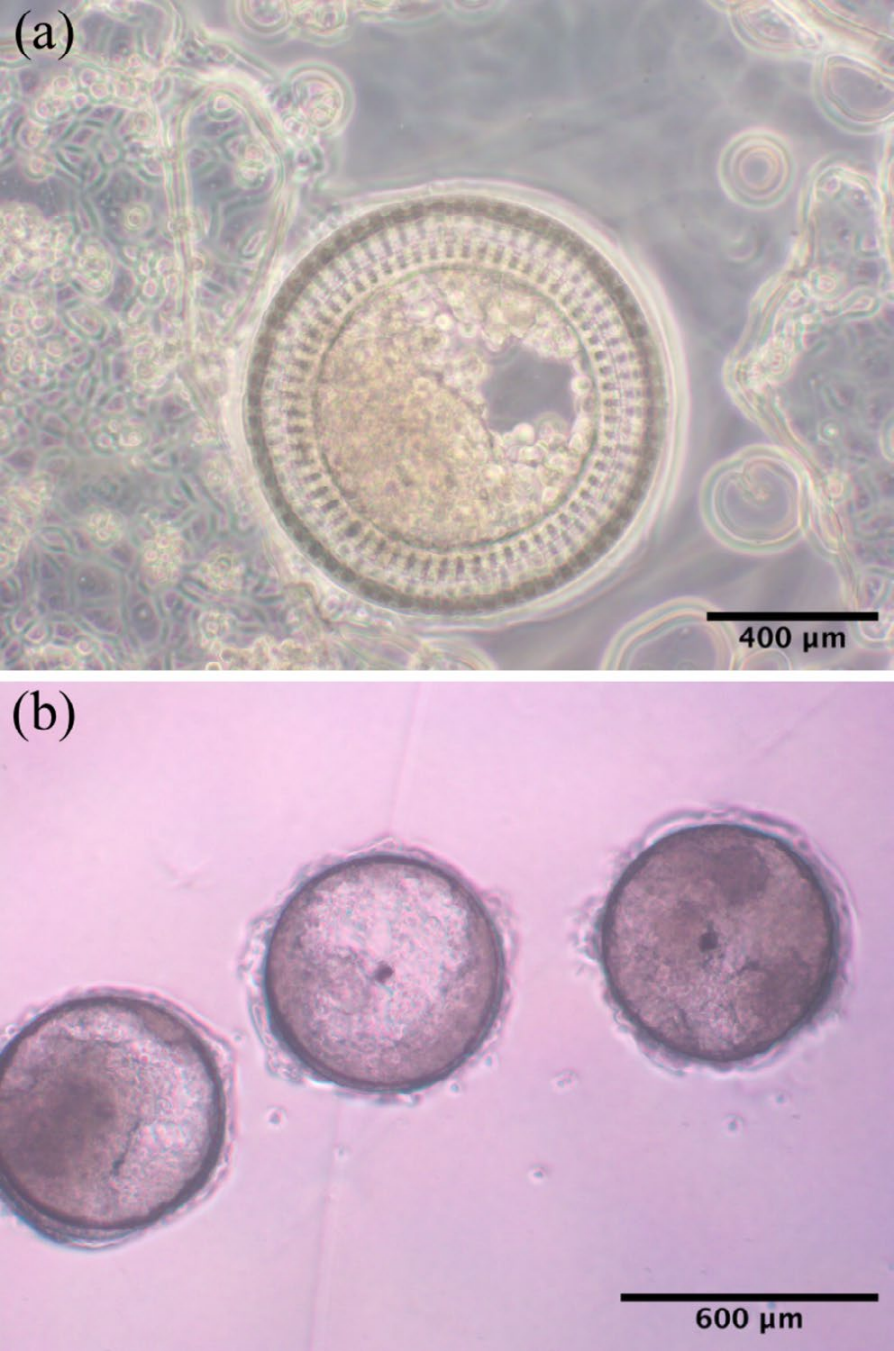
Optical images of aggregates inside 3D‐printed truncated cone and drilled cylindrical cavities. Optical microscope images of living PANC‐1 cell aggregates in different confinement geometries. (a) 3D‐printed truncated cone (base diameter: 800 μm) containing an aggregate cultured for 9 days, with PolyHEMA visible at the edge. (b) Three mechanically drilled cylindrical cavities (diameter: 500 μm) in a PolyHEMA‐coated Petri dish containing aggregates cultured for 6 days.

**FIGURE 5:**
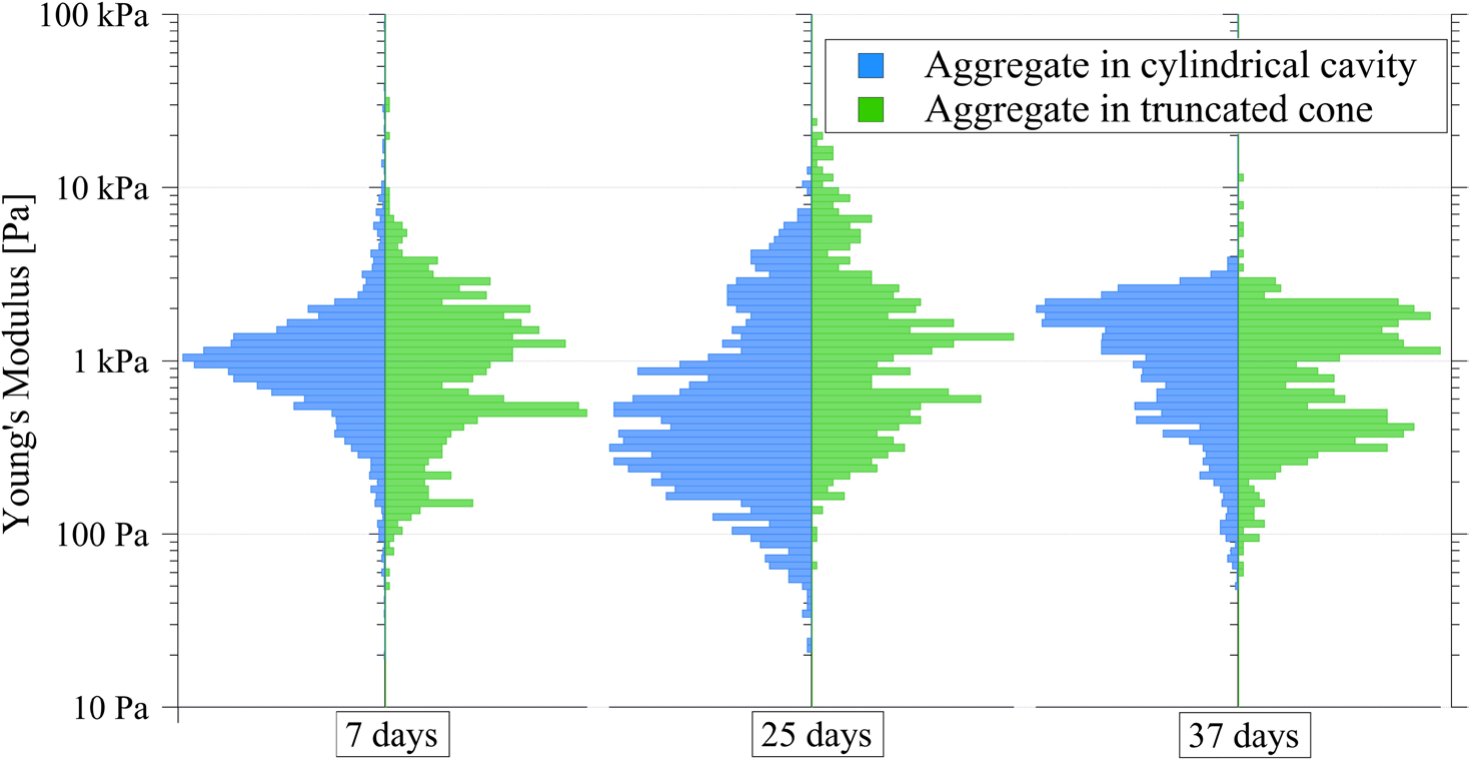
Mechanical properties of monotypic PANC‐1 aggregates confined in cylindrical cavities and truncated cones at three different time points. Violin plots show the distributions of median Young's modulus values derived from individual AFM force maps of aggregates confined within cylindrical cavities (blue) and truncated cones (green). Measurements were taken after 7, 25, and 37 days of culture, with aggregate age defined from the day of cell seeding on PolyHEMA‐coated Petri dishes.

The porous architecture of the truncated cones was designed to allow medium access within the structure, supporting prolonged cell survival. In contrast, the limited permeability of the cylindrical cavities may more closely reproduce hypoxia‐associated microenvironments typical of solid tumours. Such conditions are known to trigger adaptive cellular responses. For example, Kim et al. [39] demonstrated that three‐dimensional spheroid cultures can acquire an angiogenic phenotype mediated by fibroblast growth factor 2, mimicking in vivo adaptations to oxygen and nutrient deprivation. Comparable scaffold‐free systems, such as those generated using PolyHema‐coated dishes via the liquid overlay technique, allow the consistent formation of uniform and viable spheroids [40]. These hypoxic and diffusion‐limited conditions are particularly relevant to pancreatic ductal adenocarcinoma (PDAC), in which the dense and fibrotic extracellular matrix markedly restricts oxygen and drug delivery, thereby promoting tumour progression and therapeutic resistance [41, 42]. Figure 4 presents optical images illustrating early‐stage performance of the two confinement strategies. In Figure 4a, an aggregate cultured for 9 days is confined within a 3D‐printed truncated cone, whereas Figure 4b shows aggregates cultured for 6 days within drilled cylindrical cavities. Both confinement geometries effectively supported aggregate formation and long‐term retention during culture. Cylindrical cavities were particularly advantageous during seeding due to natural settling and reproducible positioning. Despite occasional partial detachment of the PolyHEMA coating from the ITO substrate in truncated cones, both geometries remained suitable for extended AFM studies once aggregates were stably adhered. Their porous walls were designed to facilitate medium access within the structure, which may contribute to cellular maintenance during long‐term culture. Both confinement geometries permitted monitoring of aggregates for up to 37 days after seeding. The distributions of Young's modulus obtained by AFM are shown in Figure 5. Truncated cones showed no statistically significant temporal differences (*p* = 0.267, 0.713, 0.161), whereas cylindrical cavities exhibited significant temporal variation (*p* = 1.13 × 10^−5^, 0.184, 4.28 × 10^−8^), indicating geometry‐dependent mechanical adaptation over time (see Table 2).

**TABLE 2:** Young's modulus (*E*, Pa) of single adherent cells, and confined aggregates in truncated cones and cylindrical cavities.

Sample	*n*	25th percentile	Median	75th percentile
Single cells	198	1219.84	**1874.28**	2753.84
Truncated cones	45	496.38	**1131.56**	1761.80
Cylindrical cavities	487	429.94	**753.40**	1249.13

*Note:* Median values (in bold) and 25th–75th percentiles are reported for each sample type. The number of measurements (number of force maps) per group is indicated by *n*.

## 4 Discussion

As established in earlier mechanical characterisations of multicellular systems, the progressive transition from a single‐cell state to a highly organised three‐dimensional architecture dictates macroscale tissue properties independently of physical boundary constraints [43]. Our findings indicate that the mechanical behaviour of PANC‐1 aggregates is primarily governed by this transition from single‐cell to multicellular organisation, rather than by confinement geometry. This transition represents a fundamental shift from a regime dominated by individual cortical tension and focal adhesions to one defined by collective mechanics emerging from a percolated network of intercellular junctions. In isolated cells, actomyosin‐driven tension and substrate anchoring result in a comparatively high‐stiffness profile, a mechanical state driven by individual focal adhesion dynamics and broad cytoskeletal remodelling [44]. In contrast, within aggregates, these anchoring points are largely superseded by cell–cell connectivity, leading to redistribution of internal stresses and a subsequent reduction in effective macroscale stiffness. This mechanical decoupling from the substrate aligns with previous observations in which aggregate formation was associated with reduced apparent stiffness compared with adherent single cells.

The observed increase in viscoelastic behaviour further supports the emergence of collective mechanical properties within multicellular assemblies, where externally applied stresses can be dissipated through intercellular rearrangements, heterogeneous cytoskeletal organisation, and junctional deformation. Crucially, the absence of sustained statistically significant differences between aggregates confined in truncated cones and cylindrical cavities suggests that, once a multicellular network is established, the bulk mechanical response becomes largely insensitive to external geometric constraints within the tested regime. We propose that this reflects mechanical screening within densely packed cellular assemblies, whereby deformation is redistributed internally rather than dictated primarily by boundary geometry. Consequently, although confinement shape may influence cells located near the aggregate boundaries, the aggregate interior, and the accessible surfaces probed by AFM, exhibit comparatively geometry‐independent mechanical behaviour.

Temporal differences observed specifically in cylindrical cavities further suggest that geometry influences the kinetics of mechanical adaptation rather than the steady‐state mechanical regime. We hypothesise that this behaviour may arise from geometry‐dependent variations in local transport conditions and stress accumulation. The porous architecture of the truncated cones was designed to permit greater medium accessibility within the confined environment, whereas the more restricted cylindrical geometry may favour the development of local gradients in oxygen, nutrients, or mechanical stress, as commonly observed in multicellular spheroid systems. Although these parameters were not directly quantified in the present study, they remain a plausible explanation for the observed time‐dependent variability in viscoelastic properties. Furthermore, the higher mechanical heterogeneity observed in truncated cones may reflect spatially distinct contributions from the extracellular matrix, including collagen I‐rich regions, together with confinement‐induced variations in cytoskeletal organisation [45, 46].

The two confinement strategies nevertheless differed substantially in experimental throughput and implementation. Cylindrical cavities provided a robust, reproducible, and comparatively high‐throughput platform, allowing measurements across a broader range of culture times and enabling longitudinal characterisation for up to 37 days after seeding. In contrast, the 3D‐printed truncated cones enabled controlled spatial confinement but required manual transfer of aggregates and more complex fabrication procedures, limiting throughput and increasing sample loss during handling. The transfer procedure could also occasionally disturb the PolyHEMA coating surrounding the structures, potentially contributing to local variability in aggregate positioning and organisation. For instance, when aggregates were introduced into truncated cones, partial detachment of the PolyHEMA coating from the ITO substrate was occasionally observed, as seen in Figure 4a. This may be attributed to weaker adhesion between the PolyHEMA and ITO, as under physiological conditions PolyHEMA is negatively charged due to its hydroxyl groups, while ITO also carries a negative surface charge [47]. The resulting electrostatic repulsion between these surfaces promotes local delamination. As a result, small regions of the coating may detach from the substrate. In contrast, stronger adhesion is maintained on non‐polar polystyrene Petri dishes, where PolyHEMA interacts more favourably with the substrate [48]. Despite these effects, AFM measurements were performed locally on accessible peripheral regions of the aggregates under identical indentation conditions across all systems, ensuring that the Young's modulus estimation was not influenced by central‐region artefacts. To ensure balanced comparisons between confinement geometries, the primary analysis was restricted to directly comparable time points, and peripheral measurements were used consistently, with multiple positions treated as technical replicates to assess measurement reproducibility rather than spatial heterogeneity.

From Figure 3b, it can be seen that the mean logarithms of both the storage and loss moduli are higher in single cells compared to cells in aggregates. This can possibly be explained by higher stresses due to stress fibres and adhesion sites, which are more prominent in adherent cells. The differences between aggregates in different confinements are less pronounced. In all three groups, the orientation of the principal axes of the PCA ellipses is very similar, suggesting that the underlying physics governing the correlation between storage and loss moduli is consistent across all groups, despite their differing morphology and adhesion states. Thus, the simple explanation that actin filaments predominantly determine the relationship between storage and loss moduli in all three groups may be sufficient to explain the measured properties, at least as a first‐order approximation. The PCA shows that the logarithm of the storage modulus for each cell in all groups is highly correlated with the logarithm of the loss modulus. This has been observed by us in many cases, independent of cell type and status. A straightforward explanation is that the elastic modulus is mainly determined by the stiff components of the cytoskeleton, namely actin filaments and possibly microtubules. In the case of actin crosslinking molecules, especially those crosslinkers which generate forces, further stiffening is induced. The loss modulus is determined by the viscosity of the cytosol, and in particular by the drag of filaments through the cytosol. Hence in the absence of other large contributors to the loss and storage moduli, it is expected that both are highly correlated due to the dominance of actin filaments in these quantities. Rephrased, it is reasonable to investigate the correlation between storage and loss moduli, as well as possible deviations from this simplified pattern, which may indicate the presence of additional contributors that could, for instance, modify the loss modulus without significantly affecting the storage modulus. The overall macromolecular concentration (e.g., amino acids, nucleic acids, oligosaccharides, etc.) would be a potential candidate. This provides the rationale for performing PCA across different groups. Crucially, these macromolecular and structural contributors are not static, and their influence shifts as the system evolves. Long‐term mechanical characterisation is therefore particularly relevant, as multicellular aggregates undergo continuous structural and mechanical remodelling during maturation. Most AFM studies of spheroids remain limited to relatively short experimental windows due to instability and handling constraints. The confinement approaches presented here therefore provide a practical framework for monitoring temporal evolution in tumour‐like mechanical behaviour under controlled in vitro conditions while maintaining aggregate integrity over extended culture periods. From a bioengineering perspective, these findings further suggest that physiologically relevant tumour models should prioritise the regulation of cell–cell interaction networks and tissue organisation rather than confinement geometry alone. Together, these strategies broaden the applicability of AFM to multicellular systems and may facilitate future investigations into tumour mechanobiology, multicellular remodelling, and therapeutic response.

Although the present work focuses specifically on mechanical confinement of multicellullar aggregates of PANC‐1 cells, future model developments should aim to incorporate stromal and immune components, as fibroblasts‐mediated regulation of tumour growth and immune response remains a crucial complementary aspect of the tumour microenvironment [49, 50]. In this context, the biomechanical and migratory behaviours of fibroblasts underscore the importance of including these stromal elements in advanced in vitro models [51]. Integrating such components would allow the recreation of complex mechanical interactions that occur within the tumour stroma, thereby extending the physiological relevance of confinement‐based systems.

## 5 Conclusion

In this work, two complementary microconfinement strategies were established to enable reproducible atomic force microscopy (AFM) measurements of multicellular aggregates. 3D‐printed truncated conical microstructures were designed to provide controlled spatial confinement and allow medium access within the structure, while microfabricated cavities in PolyHEMA‐coated Petri dishes provided a simple and robust platform for stable aggregate confinement. Both approaches maintained aggregate integrity under standard culture conditions for extended periods and enabled reliable assessment of viscoelastic properties over time. Aggregates consistently exhibited reduced stiffness compared with substrate‐adherent single cells, consistent with a transition from single‐cell mechanical behaviour to collective, multicellular organisation. These findings demonstrate that engineered confinement platforms provide a reliable means to probe the mechanics of multicellular aggregates, thereby extending the applicability of AFM to complex three‐dimensional cell systems. Beyond methodological development, this work supports the use of controlled microconfinement systems for long‐term mechanical characterisation of multicellular aggregates, offering a framework for future studies in tumour mechanobiology and other 3D in vitro models where cell–cell organisation plays a dominant role in determining mechanical behaviour.

## Author Contributions

I.V.M.L., C.C.M., and S.G.K. performed cell culture and AFM measurements on single cells, with I.V.M.L. analysing aggregates confined in drilled cavities and S.G.K. and C.C.M. analysing aggregates confined in 3D‐printed microstructures. M.W. contributed to cell culture and developed the PolyHEMA coating protocol used in this study. C.C.M. designed the 3D confinement structures together with M.R. and S.D. W.G. fabricated the 3D‐printed microstructures, and M.J.V. supervised the microfabrication work. J.M. established the milling‐based method for drilling cavities in Petri dishes. M.R. and M.J.V. supervised the overall research. I.V.M.L. wrote the manuscript, which was reviewed by M.R. and S.D.

## Funding

This research was supported by the Coordenação de Aperfeiçoamento de Pessoal de Nível Superior—Brasil (CAPES), Finance Code 001, for the private PhD scholarship of I.V.M.L.

## Ethics Statement

The authors have nothing to report.

## Consent

The authors have nothing to report.

## Conflicts of Interest

The authors declare no conflicts of interest.

## Supporting information


**Figure S1:** AFM force curve and Hertz model fit for an individual indentation.
**Figure S2:** Power‐law analysis of viscoelastic moduli versus frequency for aggregates in truncated cone and cylindrical confinement.
**Figure S3:** AFM frequency sweep measurement and viscoelastic model fitting for a multicellular aggregate confined in a cylindrical cavity.
**Table S1:** Viscoelastic parameters for single adherent cells and confined aggregates using a power‐law structural damping model.
**Methods S1.** Cleaning of 3D‐Printed structures.
**Analysis S1.** The sweep modulation experiment.
**Analysis S2.** Estimation of uncertainties in *E*' and *E*″.

**Videos S1:** Time‐lapse dissociation of a three‐week‐old PANC‐1 multicellular aggregate on an uncoated Petri dish.

**Videos S2:** Time‐lapse dissociation of a four‐week‐old PANC‐1 multicellular aggregate on an uncoated Petri dish.

## Data Availability

The datasets generated during and analysed during the current study are available from the corresponding author on request. No custom code was used in this study.
