# Supplementary material for: 3D‐Printed Structures Versus Drilled Cavities: A Comparison of Microconfinement Methods for Rheological Characterisation of Multicellular Aggregates
Source: J Mol Recognit. 2026 Jul 2;39(4):e70040. doi: 10.1002/jmr.70040 (PMC13324969; doi:10.1002/jmr.70040)
Supplement: Supplementary file 1 — Figure S1: AFM force curve and Hertz model fit for an individual indentation. Figure S2: Power‐law analysis of viscoelastic moduli versus frequency for aggregates in truncated cone and cylindrical confinement. Figure S3: AFM frequency sweep measurement and viscoelastic model fitting for a multicellular aggregate confined in a cylindrical cavity. Table S1: Viscoelastic parameters for single adherent cells and confined aggregates using a power‐law structural damping model. Methods S1. Cleaning of 3D‐Printed structures. Analysis S1. The sweep modulation experiment. Analysis S2. Estimation of uncertainties in E' and E″. [file JMR-39-e70040-s003.docx]

**Supplementary Material**

**3D-Printed Structures versus Drilled Cavities: A Comparison of Microconfinement Methods for Rheological Characterisation of Multicellular Aggregates**

*Isis V. M. Lima^1*^, Chukwuma Chris Muoghalu^1*^, Shruti G. Kulkarni^1^, Mènie Wiemer^1^, Jonas Michalewski^1^,* Sander van den Driesche^2,3,4^*,* Wiebke Gehlken*^2,4^*, Michael J. Vellekoop*^2,3,4^*, *and Manfred Radmacher^1^*

^1^*Institute for Biophysics, University of Bremen, Otto-Hahn-Allee 1, 28359, Bremen, Germany*

*^2^Institute for Microsensors, -actuators and -systems (IMSAS), University of Bremen, Otto-Hahn-Allee 1, 28359, Bremen, Germany*

*^3^MAPEX Center for Materials and Processes, University of Bremen, Bibliothekstraße 1, 28359, Bremen, Germany*

*^4^Microsystems Center Bremen (MCB), University of Bremen, Otto-Hahn-Allee 1, 28359, Bremen, Germany*

**Corresponding author email**: [idovalem@uni-bremen.de](mailto:idovalem@uni-bremen.de)

**Supplementary Figures**


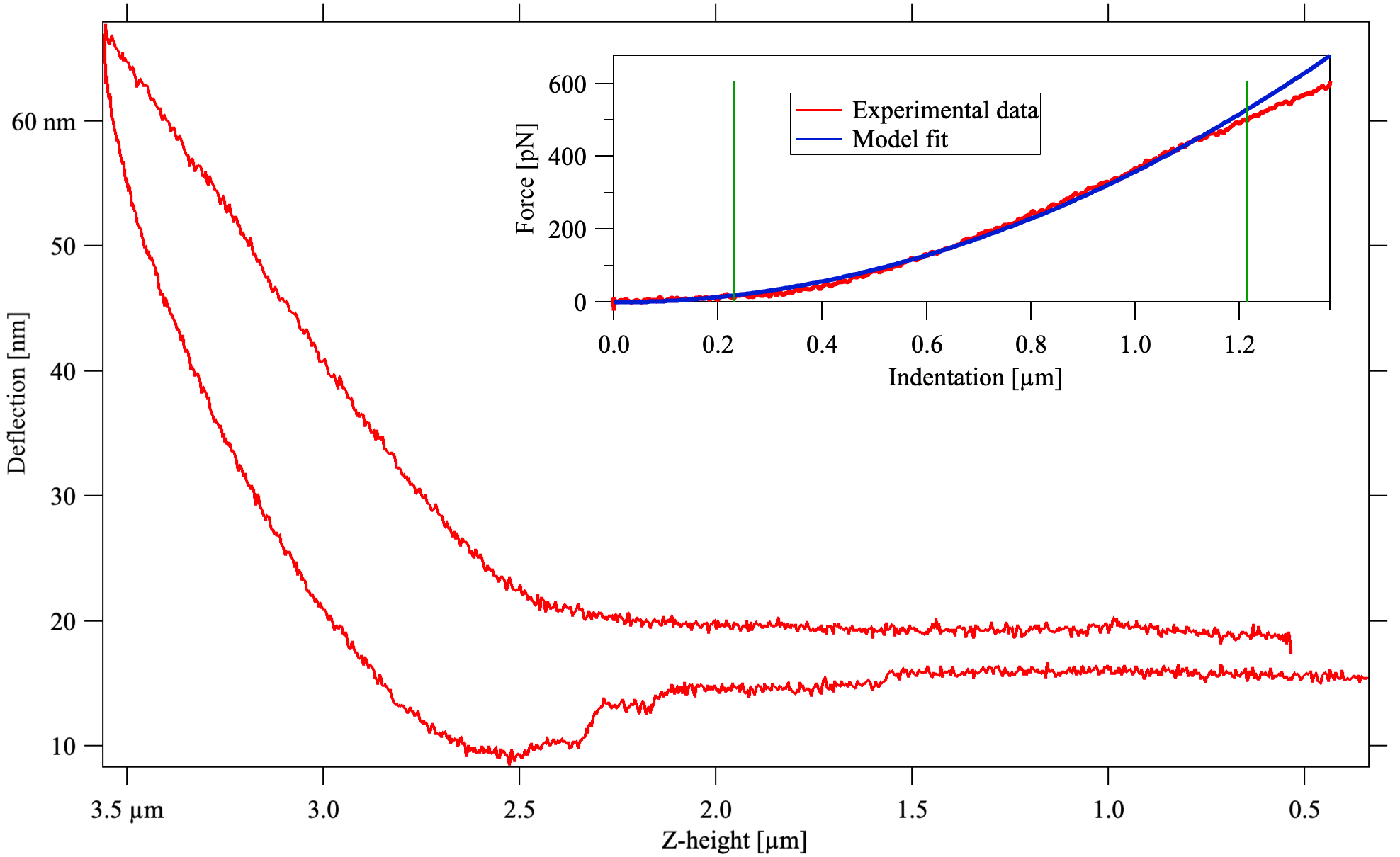


**S1. AFM force curve and Hertz model fit for an individual indentation**

Caption: An individual atomic force microscopy (AFM) force curve is presented, showing the cantilever deflection as a function of z-height (red line). The sample consisted of a multicellular PANC-1 aggregate (7 days in culture) under truncated-cone confinement. In the inset, the corresponding force-indentation data are displayed, where the experimental approach curve (red) was

fitted using the Hertz contact model (blue).



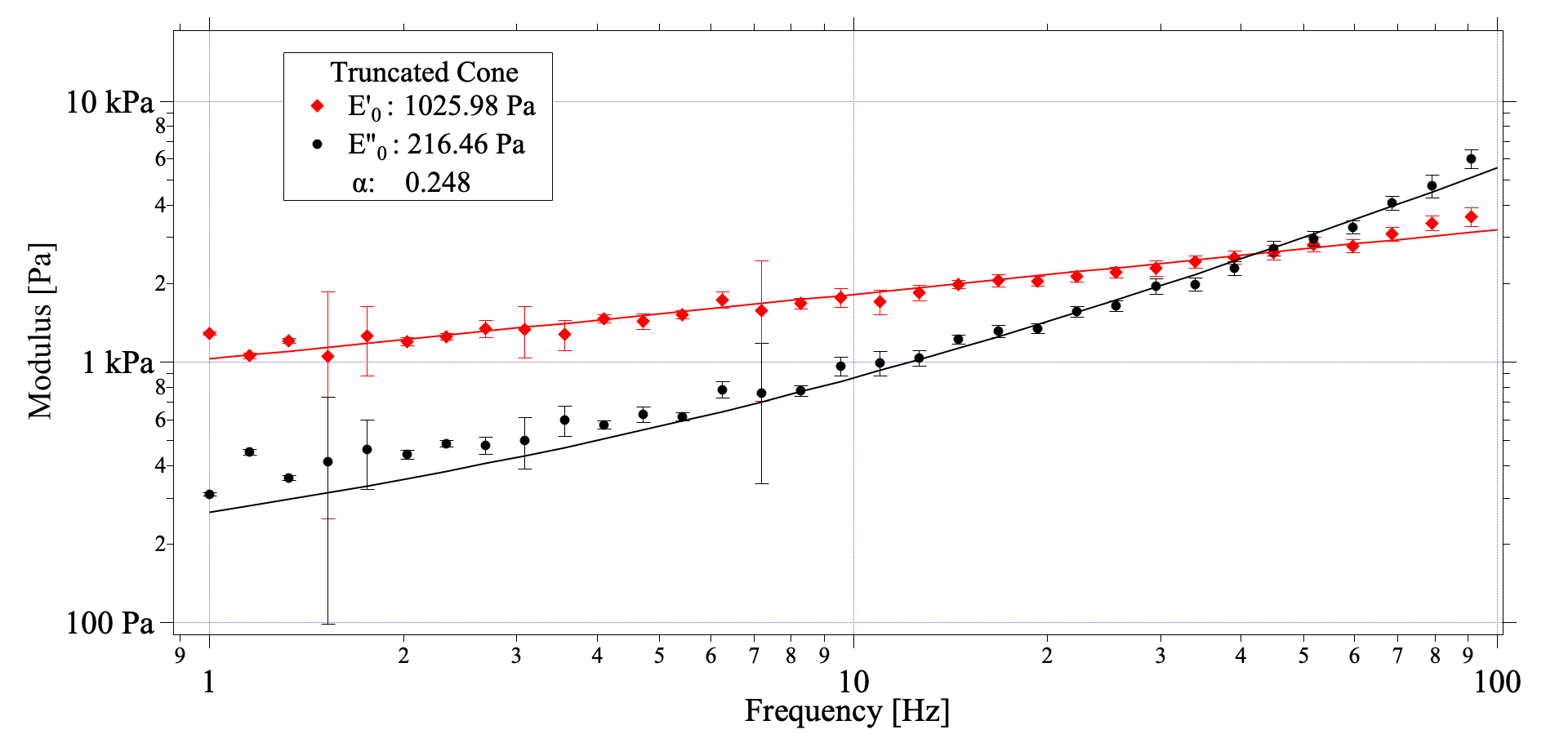
​



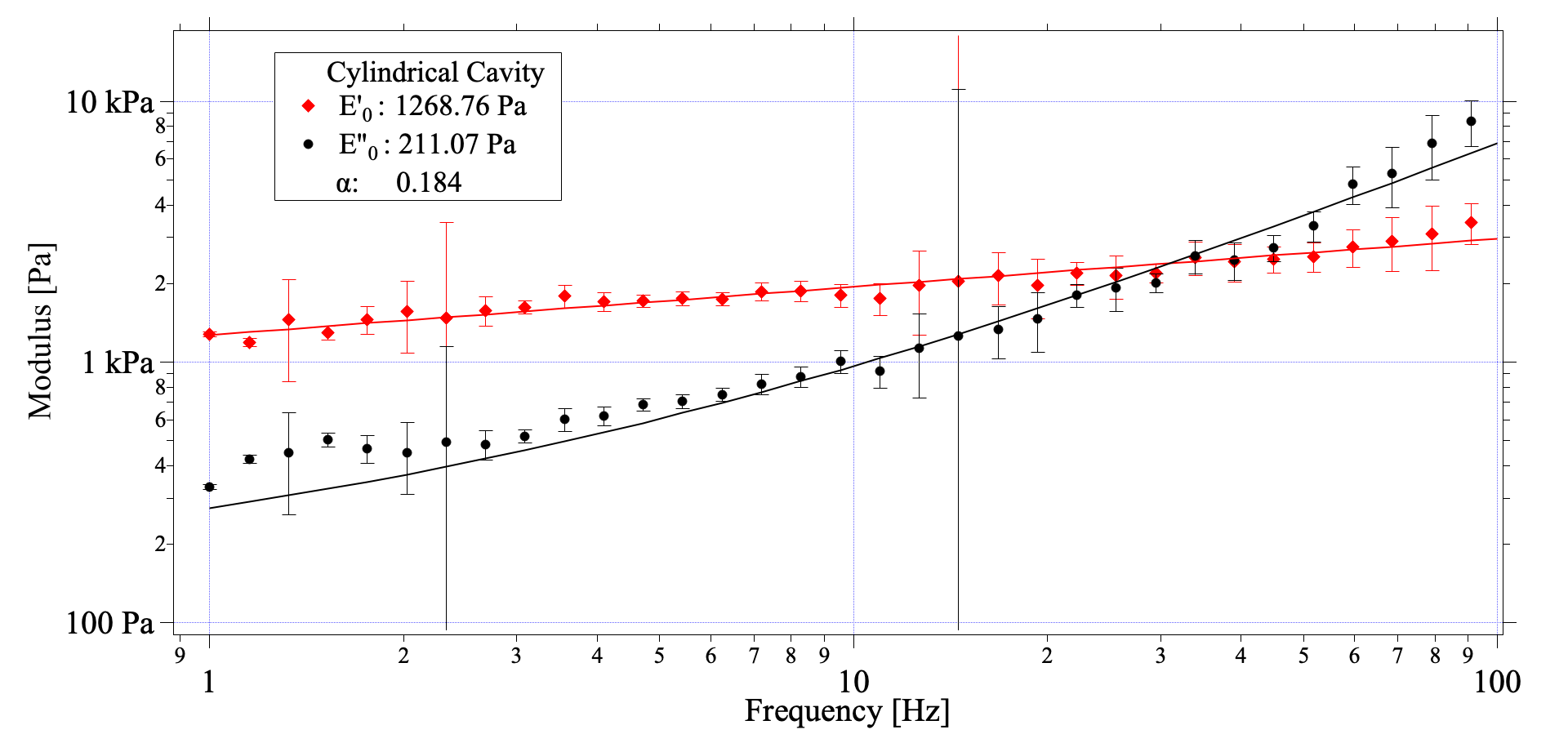
​**S2. Power-law analysis of viscoelastic moduli versus frequency for aggregates in truncated cone and cylindrical confinement.**



Caption: (a) PANC-1 aggregate cultured for 7 days in a truncated cone confinement (α = 0.248), and (b) aggregate cultured for 7 days in a cylindrical cavity confinement (α = 0.184). The graphs show representative individual force-curve measurements acquired in frequency sweep mode for each condition. The storage modulus (E′) and loss modulus (E″) were analysed using a power-law structural damping model over the measured frequency range, where α denotes the power-law exponent describing the frequency dependence of the viscoelastic response. For each frequency, corresponding values of E′ and E″ were obtained. The parameters reported correspond to the reference moduli E′₀ and E″₀, defined at a reference frequency of 1 Hz. The storage modulus data (red) exhibit a clear power-law dependence across the analysed frequency range, whereas the loss modulus data (black) also follow the expected power-law trend, with slight deviations at lower frequencies leading to curvature in the fitted response. Such behaviour is commonly observed in biological viscoelastic systems and likely reflects additional dissipative and relaxation processes not fully captured by a single power-law structural damping model.


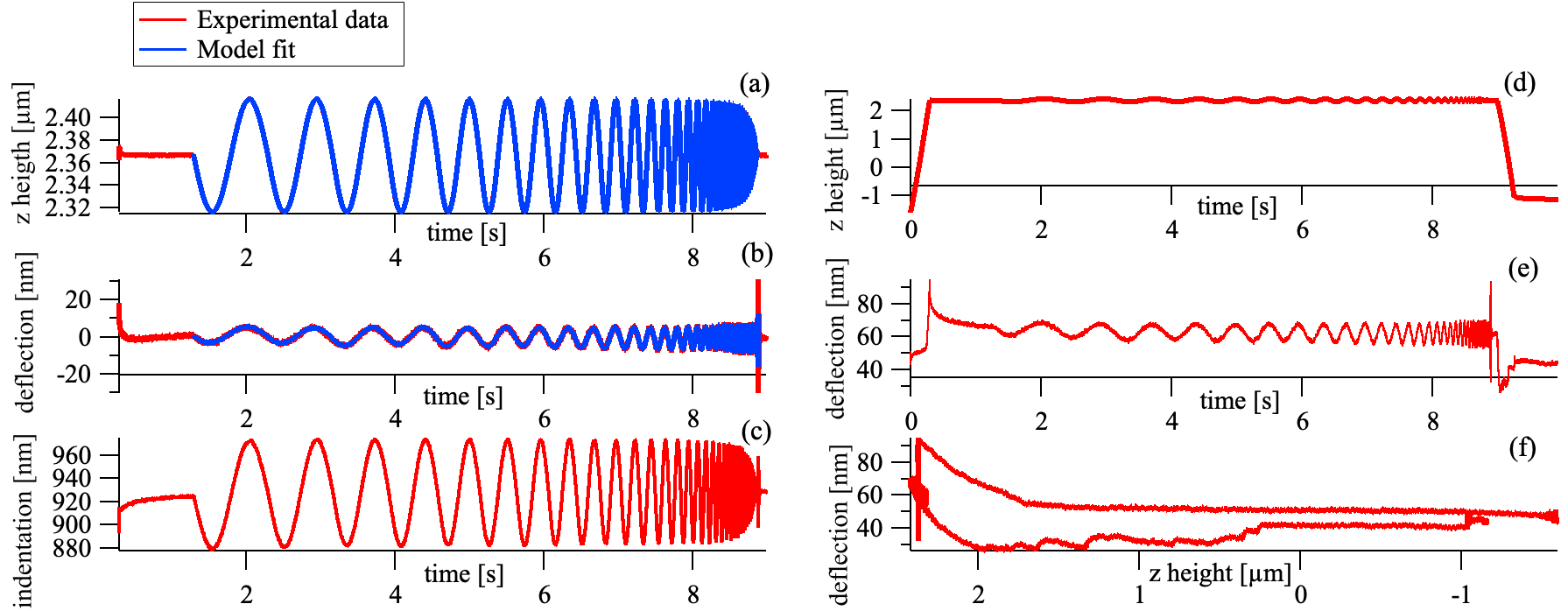


**S3. AFM frequency sweep measurement and viscoelastic model fitting for a multicellular aggregate confined in a cylindrical cavity.**

Caption: Oscillatory indentation (50 nm amplitude) was applied over 8.7 s. Experimental data (red), and model fits (blue, power-law structural damping model) are shown as (a) z-piezo displacement and (b) cantilever deflection versus time; (c) indentation depth versus time. Panels (d–f) show raw experimental signals without fitting: (d) z-piezo displacement versus time, (e) cantilever deflection versus time, and (f) cantilever deflection versus z-piezo displacement.

**Supplementary Videos**

**S1. Time-lapse dissociation of a three-week-old PANC-1 multicellular aggregate on an uncoated Petri dish.**

**S2. Time-lapse dissociation of a four-week-old PANC-1 multicellular aggregate on an uncoated Petri dish.**

Caption: Multicellular aggregates were generated and cultured on PolyHEMA-coated Petri dishes in DMEM supplemented with 10% FBS and 1% penicillin–streptomycin. Aggregates were maintained for either three or four weeks within the same culture system. At the respective time points, aggregates were taken from the same PolyHEMA-coated Petri dish culture and transferred to uncoated Petri dishes containing fresh complete medium (DMEM supplemented with 10% FBS and 1% penicillin–streptomycin). Time-lapse imaging was performed over 72 h to monitor post-transfer behaviour, including adhesion, dissociation, and cell spreading, as well as to assess sustained cellular activity. Images were acquired every 10 min (432 frames in total) using a ZEISS 4× objective (Carl Zeiss, Germany) coupled to an APTINA CMOS industrial digital camera (8 MP) mounted on an Axiovert 25 inverted microscope. Acquisition conditions were maintained at 37 °C and 5% CO_2_​, with light intensity set to 4, exposure time of 30 ms, and exposure target of 55.

**Supplementary Table**

| **‍Sample** | **n‍** | ***E^’^* (Pa)** | ***E*^’’^ (Pa)** | ***α*** |
| --- | --- | --- | --- | --- |
| ‍single cell | 761‍ | 1879.87‍ | 134.32 | 0.16 |
| ‍truncated cone | ‍238 | 1078.36‍ | 124.32 | 0.21 |
| cylindrical cavity | ‍2101 | 986.62 | 83.36 | 0.20 |

**Supplementary Table S1**. **Viscoelastic parameters for single adherent cells and confined aggregates using a power-law structural damping model.**

Caption: Comparison of viscoelastic parameters between single adherent cells and multicellular aggregates formed under distinct confinement geometries. The storage modulus (*E^’^*), the loss modulus (*E^’’^*), and the power-law exponent (*α*) were obtained by fitting the data to a power-law structural damping model. Data represent mean values from *n* independent force maps.

**Supplementary Methods**

**S1. Cleaning of 3D-Printed Structures**

To enable the reuse of 3D-printed conical microstructures previously coated with Poly(2-hydroxyethyl methacrylate) (PolyHEMA) and used for cell culture, a cleaning protocol was applied. Proper cleaning preserves the structural integrity of the microstructures and provides the advantage of repeated use, thereby reducing fabrication effort and material costs. The cleaning procedure was performed as follows. The cell culture medium was first discarded into liquid waste, and the conical microstructures were subsequently rinsed with 4 mL of Milli-Q (MQ) water, which was also discarded into the same waste container. Following this, 6 mL of a 5% Helizyme (B. Braun, Germany) working solution was added, and the conical microstructures were incubated for at least 2 h, or overnight, in a fume hood. The solution was then discarded into non-halogenated liquid waste. Afterward, the microstructures were rinsed again with 4 mL of MQ water. For sterilisation, 6 mL of 70% ethanol (EtOH) was added, followed by incubation at room temperature for 2 h in a covered container. The solution was discarded, and a second incubation was performed with 6 mL of 95% EtOH for 2 h at room temperature. Finally, the microstructures were rinsed a final time with 4 mL of MQ water. Throughout this procedure, the 3D-printed conical microstructures remained intact, although minor displacement within the substrate was occasionally observed.

**Supplementary Data Analysis**

## **S1. The sweep modulation experiment**

In a force curve,*z*is ramped from an out-of-contact position until the tip reaches the sample. We denote *z* as the measured height of the z-piezo, obtained using the built-in position sensor of the instrument.In a force curve, z is ramped from out of contact until the tip reaches the sample. We denote with z the measured height of the z-piezo using the built-in position sensor of our instrument. At some given z-height (*z_0_*)*,* the sample is touched and the deflection will have reached a value of d_0_. The contact point is determined by applying the appropriate contact model to the approach data, as described in the main text, to obtain the aApparent Young's modulus of the approach curve. At some point a defined position, the z movement is stopped at $\underline{z}$. The deflection signal will change due to creep of the cell for some time. Typically, after approximately 1 second, this creep will be reasonably constant at a value $\underline{d}$. A modulation of *z*, applied around this position $\underline{z}$ during sweep modulation, will results in a sinusoidal response in the deflection signal around $\underline{d}$. During modulation, the z-signal applied and the deflection response can be written as:

$z(t)=\underline{z}+Z*sin(\omega t)$, (1)

$d(t)=\underline{d}+D*sin(\omega t+\varphi)$. (2)

Here $\omega$ represents the frequency of the modulation and $\varphi$ is the phase shift between the drive signal (*z*) and response (*d*). In this and the subsequent expressions, the offsets (i.e., the *z* position at contact *z_0_* and the deflection value of the free cantilever *d_0_*) have already been subtracted. More precisely, the offsets of *z* and *d* signals have been adjusted to be the same as at contact *z_0_ = 0* and *d_0_ = 0*.

The indentation δ can be calculated as the difference between z-height and deflection:

$\delta(t)=z(t)-d(t)=\underline{z}-\underline{d}+Z*sin(\omega t)-D*sin(\omega t+\varphi)$, (3)

$\delta(t)=\underline{z}-\underline{d}+Z*sin(\omega t)-D*sin(\omega t)*cos(\varphi)-D*sin(\varphi)*cos(\omega t)$, (4)

$\delta(t)=\underline{z}-\underline{d}+\left( Z-D*cos(\varphi) \right)*sin(\omega t)-D*sin(\varphi)*cos(\omega t)$. (5)

The indentation can also be written as:

$\delta(t)=\underline{\delta}+\Delta*sin(\omega t+\theta)$, (6)

where $\underline{\delta}$ is the average indention, Δ is the amplitude of modulation of the indentation, and φ is the phase shift between drive (*z*) and indentation. Equation 6 can be rearranged to obtain a relationship with Eq. 5, yielding:

$\delta(t)=\underline{\delta}+I*cos(\theta)sin(\omega t)+\Delta*sin(\theta)cos(\omega t)$ (7)

$\Delta*cos(\theta)=Z-D*cos(\varphi)$ (8a)

$\Delta*sin(\theta)=-D*sin(\varphi)$ (8b)

Dividing Eq. 8b by Eq. 8a, we obtain a relation for tan($\theta$), whereas taking the sum of the squares of both equations yields a relation for the amplitude of indentation *I*:

$tan(\theta)=\frac{-D*sin(\varphi)}{Z-D*cos(\varphi)}$ (9a)

$\Delta^{2}=\left( Z-D \right)^{2}+D^{2}$ (9b)

Hertzian contact mechanics can be written depending on the tip geometry generalized as suggested by de Sousa *et al*. (2017):

$F=\frac{A_{\lambda}*E}{1-\upsilon^{2}}\delta^{\lambda}$, (10)

where F is the loading force, E the elastic modulus, ν the Poisson ratio and δ the indentation.

The power-law exponent depends on the tip geometry. Special cases are: λ =1 corresponds to a flat punch, λ =3/2 for a spherical or parabolic indenter, where λ =2 corresponds to conical or pyramidal indenters. The geometry dependent pre-factor A_λ_ can be seen from the above formulas and can be written as $\frac{4}{3}*\sqrt{R}$ for the case of a parabolic indenter, or as $\frac{1}{\sqrt{2}}tan\alpha$ in the case of a 4-sided pyramid. Although it is labelled with the index λ, it is not a function of λ. (Nota bene: this nomenclature has limitations, since some geometries, mainly cone vs 3- or 4-sided pyramids, have the same power-law exponent, but different prefactors. For the sake of simplicity, we refrain from introducing an additional index µ, which would run through all models, with a corresponding power-law exponent λ_µ_ and a prefactor A_µ_, as this would complicate the notation without providing significant benefit). The indentation in Eq. 7 is time-dependent, as given by Eq. 6 due to the modulation. Thus, we obtain:

$F=\frac{A_{\lambda}*E}{1-\nu^{2}}\left( \underline{\delta}+\Delta*sin(\omega t+\theta) \right)^{\lambda}$ (11)

$F=\frac{A_{\lambda}*E}{1-\nu^{2}}{\underline{\delta}^{\lambda}\left( 1+\frac{\Delta}{\underline{\delta}}*sin(\omega t+\theta) \right)}^{\lambda}$ (12)

When expanding the indentation in Eq. 10 which is valid if the amplitude of indentation is small compared to the average indentation $\underline{\delta}$, we get:

$F=\frac{A_{\lambda}*E}{1-\nu^{2}}\underline{\delta}^{\lambda}\left( 1+\lambda\frac{I\Delta}{\underline{\delta}}*sin(\omega t+\theta) \right)$ (13)

$F=\frac{A_{\lambda}*E}{1-\upsilon^{2}}\underline{\delta}^{\lambda}+\lambda\frac{A_{\lambda}*E}{1-\nu^{2}}\underline{\delta}^{\lambda-1}\Delta*sin(\omega t+\theta)$ (14)

$F=\frac{A_{\lambda}*E}{1-\upsilon^{2}}\underline{\delta}^{\lambda}+\lambda\frac{A_{\lambda}*E}{1-\nu^{2}}\underline{\delta}^{\lambda-1}\Delta*sin(\omega t+\theta)$ (15)

The first term is the average force, whereas the second term can be separated in an in-phase and out-of-phase part.

$F=\underline{F}+\lambda\frac{A_{\lambda}*E}{1-\upsilon^{2}}\underline{\delta}^{\lambda-1}\Delta*\left( sin(\omega t)*cos(\theta)+cos(\omega t)*sin(\theta) \right)$ (16)

$F-\underline{F}=\lambda\frac{A_{\lambda}*1}{1-\upsilon^{2}}\underline{\delta}^{\lambda-1}\Delta*\left( sin(\omega t)*E*cos(\theta)+cos(\omega t)*E*sin(\theta) \right)$ (17)

From the above equation, we can assign the storage modulus E' and the loss modulus E'' in the following way:

$E'=E*cos(\theta)$ (18a)

$E''=E*sin(\theta)$ (18b)

The oscillating component of the force is given by the cantilever's force constant times the deflection signal amplitude Eq. 2, which can also be split in the in phase and out of phase part:

$F-\underline{F}=k_{c}*D*sin(\omega t+\varphi)=k_{c}D*\left( sin(\omega t)cos(\varphi)+cos(\omega t)sin(\varphi) \right)$ (19)

Comparing Eq. 19 with Eq. 17 and splitting the in phase and out of phase parts, we get the following two relations:

$k_{c}D*cos(\varphi)=\lambda\frac{A_{\lambda}*E}{1-\upsilon^{2}}\underline{\delta}^{\lambda-1}\Delta*cos(\theta)=\frac{{\lambda A}_{\lambda}*1}{1-\nu^{2}}\underline{\delta}^{\lambda-1}\Delta*E'$, (20a)

$k_{c}D*sin(\varphi)=\lambda\frac{A_{\lambda}*E}{1-\upsilon^{2}}\underline{\delta}^{\lambda-1}\Delta*sin(\theta)=\lambda\frac{A_{\lambda}*1}{1-\nu^{2}}\underline{\delta}^{\lambda-1}\Delta*E''$. (20b)

This gives the final expressions for the storage modulus (E') and the loss modulus (E'') as functions of the measurable quantities (D, Δ, and φ), as given by Eq. 9b:

$E'=\frac{\frac{D*k_{c}}{\left( 1-\nu^{2} \right)}*1}{{{\lambda*A}_{\lambda}\underline{\delta}}^{\lambda-1}\Delta}cos(\varphi)$, (21a)

$E''=\frac{\frac{D*k_{c}}{\left( 1-\nu^{2} \right)}*1}{{{\lambda*A}_{\lambda}\underline{\delta}}^{\lambda-1}\Delta}sin(\varphi)$. (21b)

The loss and the storage modulus are a function of frequency and in the simplest case we expect power law behaviour. However, in our case at frequencies beyond 10 Hz (depending on experimental parameters especially dimension of the cantilever) hydrodynamic drag of the cantilever will become very prominent. Thus we employ the structural damping model as has been introduced to AFM rheology by (Alcaraz *et al.*, 2002). Here, the complex modulus E^*^ is written as:

$E^{*}\left( \omega\right)=E_{0}*\left( 1+i\eta\right)*\left( \frac{\omega}{\omega_{0}} \right)^{\alpha}+i\mu\frac{\omega}{\omega_{0}}$, (22)

where *E_0_* is the absolute value of the modulus, *i* is the imaginary unit, *η* is the ratio between loss modulus and storage modulus (often called the loss tangent), *ω_0_* is the frequency scale (in our case we use 1Hz), *α* is the power-law exponent of the sample, and *µ* is the strength of the hydrodynamic damping, which depends on the shape of the cantilever and the viscosity of the medium.

**S2. Estimation of Uncertainties in E' and E''**

In sweep mode, the z-signal is modulated for one period at a given frequency before proceeding to the next frequency. The frequencies follow a geometric series, resulting in a constant spacing on a logarithmic scale. In our case, due to technical limitations of the instrument, the piezo is driven over a frequency range from 1 Hz to 1 kHz using 17 different frequency values per decade. For each cycle, a sinusoidal function is fitted to the deflection data in order to determine the force amplitude and phase shift, as well as to the indentation data. The latter is calculated from the measured *z* and *d* data. This procedure allows the amplitude and phase of the indentation at this frequency to be determined. In addition, the uncertainties in the directly determined quantities (D, Δ, and φ) are obtained from the covariance matrix of the respective fits. The errors in the storage and loss moduli (*E'* and *E''*) are then determined using Eqs. 21a and 21b, following standard error propagation procedures. These uncertainties are represented as error bars in the power-law plot *Supplementary Material [Supplementary Figure S2]*. At this stage, errors in the signals themselves are neglected. The noise in the z-sensor signal is very small compared with the modulation amplitude and can therefore be ignored. The noise in the deflection signal is approximately ±0.5 nm peak-to-peak, corresponding to an rms error of about 5% relative to the modulation amplitude (±5 nm in this case). This value depends on the stiffness of the sample. These fluctuations do not represent instrumental error in the classical sense, but arise from thermal fluctuations of the cantilever. As such, they are intrinsic and cannot be eliminated. Consequently, they should not be regarded as measurement error, but rather as a physical contribution to the signal. Nevertheless, they introduce additional uncertainty in *E′* and *E′′*, which is estimated to be on the order of 2%.
